# Sources of Hospital Revenne

**Published:** 1894-01-06

**Authors:** 


### HOSPITAL FINANCE.

### I.—SOURCES OF HOSPITAL REVENUE.

The system of management adopted bv any public institution is largely modified by the source from which its funds are derived. The question of finance is a vital one, even before the first stone is laid. Not merely must the money be had; it must be obtained from the right people, and collected in the right way, or there will be an almost certain falling away from the highest purpose and efficiency of the proposed work. In hospital work the effects of the administration extend to every member of the community, whether directly benefited or no, and the financial aspect becomes one of tremendous importance. The educational force implied, both in the getting together and the expenditure of the vast sums annually provided for hospital work, can hardly be overestimated, and the results for good and evil on donors, recipients, and officials can be clearly read in the history of hospital progress before us Perhaps in time it will dawn upon the managers of charitable institutions that the elaborate instruction in the art of begging imparted by their system of begging cards and street collections is not the most rational method to adopt for relieving distress largely due, in fact, to this fatal habit of depending on other people's purses. The old-fashioned delicacy which shrank from any personal appeal for money is being strangely educated out of the modern child, who is taught to worry friend and stranger alike for purposes least concerning them.

The main sources of hospital income are: (1) The State; (2) the municipality or commune; (3) endowments; (4) voluntary subscriptions and donations; (5) patients' payments.

It is seldom, however, that an institution depends on any one of these sources alone, and the extent to which one or other predominates varies according to the country, period of foundation, and general character of the work carried on.

1. In no country is the hospital revenue supplied exclusively by the State. In Norway and Sweden, where the system of State relief may be considered almost ideally perfect, the State hospitals are to a great extent self-supporting. The directors of the Christiana Hospital annually submit for the consideration and approval of Parliament a budget in accordance with a calculation based upon the number of patients, the number of beds occupied, and the daily payment of each patient during the previous year. Any deficit is voted annually by the National Parliament. The extreme poor, who are unable to make any contribution towards the expense of their treatment, are provided for by co-operation between the Government and the poor-law authorities, by which the latter pay a fixed sum for any poor-law case treated in the hospital. The scale of payment is, for the first-class, or pauper patients, 3s. a day; for the second-class, 3s. 8d. a day; for the third-class, who have separate rooms and special attention and nursing, 6s. 8d. a day. The hospital tax in Norway averages something over £34,000 a year. The remaining charges, amounting to about twice as much again, are met by the payments of patients, by the interest on endowments, and by grants from the communes.

Oddly enough it is in the British colonies that the principle of State supported hospitals has been most fully developed. Many of the colonial hospitals have indeed been provided and endowed by private individuals, but in almost every case it has become necessary to call in the aid of a Government subsidy towards the maintenance, and the extraordinary rapidity with which in many places the population has increased, has rendered it imperatively necessary for the Government to undertake the duty of providing hospital accommodation—a duty which could only be performed gradually or spasmodically by private effort. The proportionate amount contributed by the State varies very much in the different colonies, as may be seen from the following recent statistics of hospital revenues in various parts of the world.

| | State Grant (including in some places local rate). | Income from Other Sources (including patients' payments). |
|---|---|---|
| British Guiana | $107,967 | $4,282 |
| Ontario | $67,000 | $121,000 |
| Cape Town (six hospital) | £32,101 | £15,954 |
| New South Wales | £61,399 | £71,562 |
| Victoria | £229,041 | £172,889 |
| New Zealand | £54,332 | £25,189 |

In the Australasian colonies the poor-law encroaches largely on the hospital system, but the voluntary offerings are liberal, and many of the hospitals approximate to the voluntary system of England. The Government subsidies are usually granted on the principle of a pound for a pound, the State doubling the amount collected by private charity. In the West Indies, where ready money is notoriously scarce, the amount of voluntary offerings is quite inconsiderable, and Government has almost all the work to do.

2. It is remarkable that although local support is the soundest of all incomes for hospital work when voluntarily offered, it is the origin of much internal weakness when extorted by means of a rate. The history of English poor-law infirmaries amply illustrates this. Extreme parsimony in the assignment of special accommodation for the sick, in the payment of medical officers, in the supply of necessaries, and in the provision of nurses, has been, with a few notable exceptions the rule in these establishments. And this often in localities where the grudging ratepayers have been nobly distinguished in founding voluntary institutions at enormous cost. The irresistible pressure of public opinion, as attention came to be directed more generally to the condition of the sick poor, has slowly set on foot a better order of things, and stamped out many flagrant abuses. But the bitter hatred of the sick wards in workhouses still entertained in most places by the respectable poor proves that much still remains to be done. And the poor rate is no more popular than ever. In the United States of America provision is made for the sick of each town by the municipal authorities. As in England, the rate-supported hospitals are largely supplemented by voluntary effort, and it would be interesting to learn what standard of efficiency they attain compared with the distinctly charitable institutions. Some light is thrown on the matter, tending to show that human nature is much the same on the other side of the Atlantic, by the statement before us that "the cost in municipal hospitals is, as a rule, less by fifty per cent. than it is in those maintained by endowment or voluntary aid." The inference is obvious.

In Belgium, where the communal system, or the principle of the responsibility of each locality for its own sick, is most fully developed, the danger of excessive economy on the part of the local authorities is minimised by the existence of a common fund of rich endowments. "The property of all the institutions is amalgamated into one joint patrimony, in accordance with the laws relating to the organisation of public charities dating back to the year 1794. In March, 1876, a law was made by which the expenses of maintenance of the destitute devolve upon the charity administrations. It is only in presence of a deficiency in their resources that the commune, or if need be, the province, is appealed to for a subsidy."

(*To be continued.*)

